# Telangiectatic capillaries remodel over time in eyes with persistent diabetic macular edema

**DOI:** 10.1007/s00417-025-07080-5

**Published:** 2026-01-07

**Authors:** Yousef A. Fouad, Reem Mohsen, Raef Dimitry, Dina Baddar

**Affiliations:** 1 Ophthalmology Department, Ain Shams University Hospitals, Ramses st., Abbassiya, Cairo, 11517 Egypt; 2 Watany Eye Hospital, Cairo, Egypt; 3 Frimley Health NHS Foundation Trust, Frimley, UK; 4 Research Institute of Ophthalmology, Giza, Egypt

**Keywords:** Cystoid macular edema, Diabetic retinopathy, Telangiectatic capillaries, TelCaps

## Abstract

**Purpose:**

To study telangiectatic capillaries (TelCaps) in the context of diabetic macular edema (DME) and the response to injection therapy.

**Methods:**

A retrospective analysis of 25 eyes with center involving DME that had TelCaps diagnosed on infrared reflectance (IR) and optical coherence tomography (OCT) B-scans with at least one year of follow-up. The size of the TelCaps was assessed on OCT IR and B-scans. The main outcome measures were the change in visual acuity (VA), central subfield thickness (CST), OCT biomarkers of DME, and size and number of TelCaps.

**Results:**

Compared to baseline, at 1 year and final follow-up (mean 44 months), the mean logMAR VA changed from 0.23 to 0.26 and 0.34, respectively (*p* = 0.296 and 0.139), and the mean CST changed from 325 μm to 348 μm and 355 μm, respectively (*p* = 0.172 and 0.098). 72% of the eyes had unchanged or worse VA and 64% had increased CST on final follow-up. The mean number of TelCaps per eye decreased from 1.36 at baseline to 1.12 at 1 year and 0.76 at the final follow-up (*p* = 0.04), with disappearance in 11 eyes (44%), and new TelCaps appearing in 3 eyes (12%). The mean size of the TelCaps was also significantly reduced at 1-year and final follow-up on both OCT IR images and B-scans.

**Conclusion:**

Visualized on OCT B-scans and IR, TelCaps are dynamic structures that undergo significant remodeling over time. Eyes with DME and TelCaps treated with injection therapy of anti-VEGF or steroids after at least one year exhibited persistent DME despite a decrease in number and size of TelCaps. TelCaps may serve as a potential biomarker for chronic DME.

## Abstract (highlights)

**Key messages:**

**What is known:**

- The gold-standard for Telangiectatic capillaries (TelCaps) detection is indocyanine green angiography, which is invasive and may not be readily available in all settings.
- Diabetic macular edema (DME) associated with TelCaps may respond poorly to injection therapy by anti-vascular endothelial factor and steroids.

**What is new:**

- Infrared reflectance imaging complemented by optical coherence tomography B-scans are effective in the detection and measurement of TelCaps, which appeared to be dynamic lesions that remodel over time.
- Eyes with DME and TelCaps with at least one year of follow-up ended up with persistent edema and stable or worse vision after injection therapy.

## Introduction

The global prevalence of diabetes mellitus (DM) continues to grow [1] and diabetic eye disease is increasingly becoming a public health concern as a major cause of visual impairment and blindness among the working-age population [2]. Diabetic macular edema (DME) is the main cause of visual decline in diabetic patients [3], with an estimated prevalence of 5% among patients with DM and higher rates in low-to-middle-income countries [4]. The pathogenesis of DME involves breakdown of the blood-retinal barrier, inflammation, neurodegeneration, and Müller glia drainage dysfunction [3, 5]. Vascular endothelial growth factor (VEGF) is a key molecule in the pathogenesis of DME and the introduction of anti-VEGF injection therapy has revolutionized the management of the condition [6]. Treatment is reserved for center-involving DME that is identified on optical coherence tomography (OCT). Nevertheless, up to 40% of eyes with DME develop chronic persistent edema [7], highlighting the need for identification of imaging biomarkers that predict poor response and allow early modification of the treatment plan.

Currently proposed biomarkers for recalcitrant DME include greater baseline central subfield thickness (CST), greater number of hyperreflective foci, disorganization of the inner retinal layers (DRIL), and loss of ellipsoid zone (EZ) integrity [8, 9]. Telangiectatic capillaries (TelCaps) is a recent nomenclature given to large capillary aneurysms that develop in the setting of retinal vascular disease [10]. A contemporary report has suggested that up to two-thirds of eyes with DME may harbor TelCaps [11]. Studies of TelCaps have suggested their association with recalcitrant DME [10, 12] with variable response to anti-VEGF injections [13, 14], steroid therapy [15], or laser photocoagulation [16, 17]. In this study, we aimed to analyze the long-term follow-up of TelCaps in eyes with DME, their association with other OCT biomarkers, and the response of the associated macular edema to injection therapy.

## Methods

This was a retrospective analysis of eyes diagnosed with DME between January 2019 and June 2024 at Watany Eye Hospital, Cairo, Egypt. The study was approved by the institutional review board of Watany Research and Development Center and was conducted in accordance with the tenets of the Declaration of Helsinki. The need for patient consent was waived by the review board since this was a retrospective analysis and the data were de-anonymized.

The inclusion criteria were (1) treatment-naive eyes with center-involving DME defined as a CST outside the normal limits identified on the nine standard Early Treatment of Diabetic Retinopathy Study (ETDRS) grid, (2) presence of TelCaps adjacent to the DME identified using infrared reflectance (IR) images and confirmed by OCT B-scans (SPECTRALIS, Heidelberg Engineering, Heidelberg, Germany) in accordance to the previously published description [16] and using the most inclusive cut-off diameter of 100 μm for each individual lesion [11, 18, 19], and (3) at least one year of available follow-up clinical and imaging data. The diameter cutoff of 100 μm was applied to IR images since not all eyes had high-density OCT B-scans that necessarily crossed the center of the lesion; the B-scans were used to confirm the characteristics of the lesion: spanning the outer retinal layers, often with a hollow center and a hyperreflective wall. The exclusion criteria were (1) concurrent retinal pathology (e.g., retinal vascular occlusion, macular neovascularization, uveitis), (2) eyes with non-center-involving DME at baseline, (3) poor quality OCT B-scans or IR images that did not allow proper grading, (4) a history of pars plana vitrectomy, and (5) silent TelCaps with no associated DME.

**Fig. 1:**
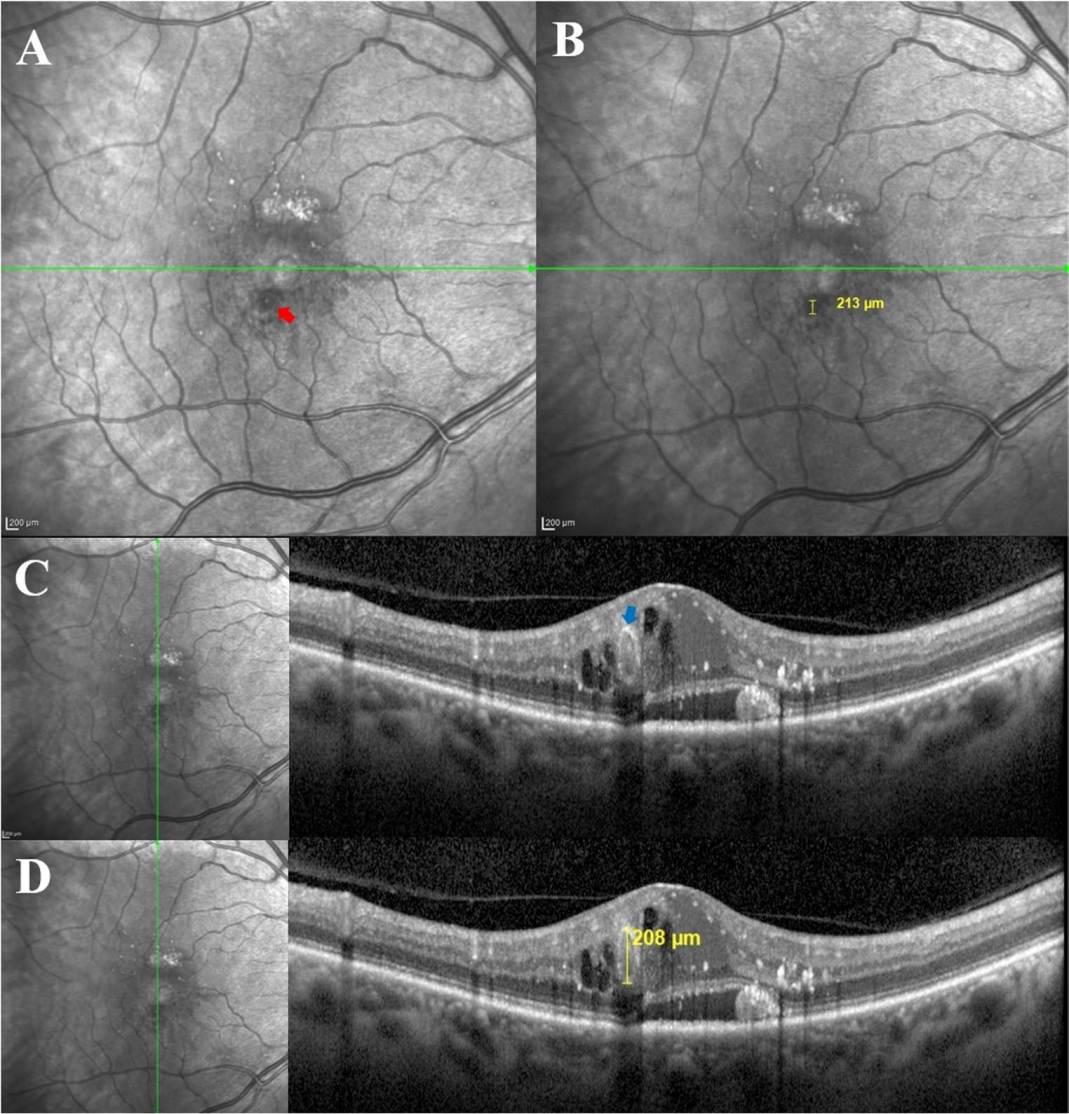
An example of identification and measurements of a telangiectatic capillary (TelCap) on infrared reflectance images (**A**, **B**) and B-scans (**C**, **D**) of optical coherence tomography. On infrared reflectance (**A**), the TelCap (red arrow) appeared as a hyporeflective circular lesion with a central hyperreflective dot in the lumen. Size measurement was done using the manual caliper tool in the widest diameter of the lesion (**B**, 213 μm). On B-scan images (**C**), the TelCap (blue arrow) appeared as an ovoid lesion within the middle retinal layers in the parafoveal area, with a well-defined hyperreflective wall and a hyporeflective lumen, adjacent to cystoid edema, hard exudates, and hyperreflective foci. A manual caliper was used to assess the widest diameter of the lesion (**D**, 208 μm)

Baseline, 1-year and final visual acuity (VA) were recorded in Snellen format and converted to the logarithm of the minimal angle of resolution (LogMAR) using standard methods [20]. Images were independently graded by 2 graders (YAF and DB), The size of the TelCaps was determined as the widest diameter of the lesion on IR images using the manual caliper tool. The size was also manually assessed in the corresponding OCT B-scans as the widest external diameter of the lesion (Fig. 1). The following biomarkers were assessed at baseline, 1-year and final follow-up: CST using the ETDRS grid, intraretinal and subretinal fluid, EZ disruption, DRIL, and HRF. The number and type of received injections were recorded.

Statistical analysis was performed using the Statistical Package for the Social Sciences (SPSS version 25). Summary descriptive statistics were expressed in terms of mean and standard deviation (SD) for quantitative variables, and in terms of percentages for categorical variables. Comparison between paired variables was made using the paired t-test for continuous variables and the Fisher’s exact test for categorical variables. A *p*-value less than 0.05 was considered statistically significant.

## Results

The final analysis included 25 eyes belonging to 21 patients. The mean (± SD) age of the patients was 63.6 (± 10) years, with a range of 38–78 years. A greater proportion of the patients were females (13 patients, 61.9%). All patients had type 2 DM with a mean duration of 22.1 (± 7.1) years.

The baseline characteristics of the study eyes are depicted in Table 1. The mean (± SD) logMAR VA was 0.23 (± 0.21) and the mean CST was 325.2 (± 60.7) µm. All eyes had intraretinal fluid and 1 eye (4%) had subretinal fluid. Six eyes (24%) had EZ disruption, and 3 eyes (12%) had DRIL at baseline. Five eyes (20%) had proliferative retinopathy and had received prior panretinal photocoagulation therapy while the remaining eyes (80%) had non-proliferative disease.

**Table 1:** Comparison of variables among study eyes (*n* = 25) at baseline, 1 year follow-up, and last visit

Variable	Baseline	1 Year follow-up	*P*-value	Last Visit	*P*-value*
LogMAR VA Mean ± SD	0.23 ± 0.21	0.26 ± 0.24	0.296^†^	0.34 ± 0.41	0.139^†^
CST (µm) Mean ± SD	325.2 ± 60.7	347.5 ± 83.2	0.172^†^	355 ± 86.3	0.098^†^
Intraretinal fluid N (%)	25 (100%)	23 (92%)	0.489^§^	22 (88%)	0.234^§^
Subretinal fluid N (%)	1 (4%)	0 (0%)	1^§^	0 (0%)	1^§^
EZ disruption N (%)	6 (24%)	7 (28%)	1^§^	10 (40%)	0.363^§^
DRIL N (%)	3 (12%)	3 (12%)	1^§^	5 (20%)	0.701^§^

The characteristics of the TelCaps are depicted in Table 2. The mean number of TelCaps per eye was 1.36 (± 1.04) with a range of 1–6. Two eyes (8%) had TelCaps within the foveal region (central 1000 μm of the ETDRS circle); in the remaining eyes (92%), the TelCaps were extrafoveal in location. The mean size of the TelCaps on IR was 173.6 (± 74.1) µm and on OCT B-scans was 92.6 (± 42.5) µm.

**Table 2:** Comparison of telangiectatic capillaries (TelCaps) characteristics among study eyes at baseline, 1 year follow-up, and last visit

Variable	Baseline	1 Year Follow-up	*P*-value	Last Visit	*P*-value*
TelCaps number Mean ± SD	1.36 ± 1.04	1.12 ± 0.88	0.829^†^	0.76 ± 0.83	**0.04** ^†^
TelCaps size IR (µm) Mean ± SD	173.6 ± 74.1	112.3 ± 89.1	< 0.001^†^	88.9 ± 90.9	**< 0.001** ^†^
TelCaps size OCT (µm) Mean ± SD	92.6 ± 42.5	71.6 ± 61	**0.045** ^†^	60 ± 67.1	**0.01** ^†^

All eyes received intravitreal injection therapy in a pro re nata (PRN) approach with a mean of 4.8 (± 4.5) injections. Thirteen eyes (52%) were treated with multiple agents. Three eyes (12%) received Bevacizumab injections, 20 (80%) received Ranibizumab injections, 10 (40%) received Aflibercept injections, 1 (4%) received Faricimab injections, and 7 (28%) received dexamethasone implants. None of the eyes received focal laser photocoagulation therapy.

The mean follow-up duration was 44.6 (± 36.8) months with a range of 12–108 months. Changes in clinical and OCT features after 1-year and on the last visit are depicted in Table 1. After 1 year and an average of 3 injections, the mean logMAR VA (0.26 ± 0.24) and CST (347.5 ± 83.2 μm) were comparable to the baseline visit (0.23 ± 0.21, *p* = 0.296, and 325.2 ± 60.7 μm, *p* = 0.172), which remained the case on final follow-up (0.34 ± 0.41, *p* = 0.139, and 355 ± 86.3 μm, *p* = 0.098). The mean change in logMAR VA was + 0.03 at 1-year and + 0.11 on final follow-up. Twelve eyes (48%) had worse VA on the final visit, 6 eyes (24%) had stable VA, and 7 eyes (28%) had improved VA. Regarding CST, the mean change was + 22.3 μm at 1 year and + 29.8 on final follow-up. 64% of the eyes had increased CST and 36% had reduced CST on final follow-up. Only 3 eyes (12%) had a dry macula on OCT on final follow-up, 4 eyes (16%) developed EZ disruption and 2 eyes (8%) developed DRIL. In the group that received intravitreal steroid implants (7 eyes), neither the VA nor the CST significantly differed on baseline and final visits (0.14 ± 0.08 vs. 0.24 ± 0.17, *p* = 0.193, and 301.4 ± 24.6 μm vs. 355 ± 61.2 μm, respectively, *p* = 0.068).

**Fig. 2:**
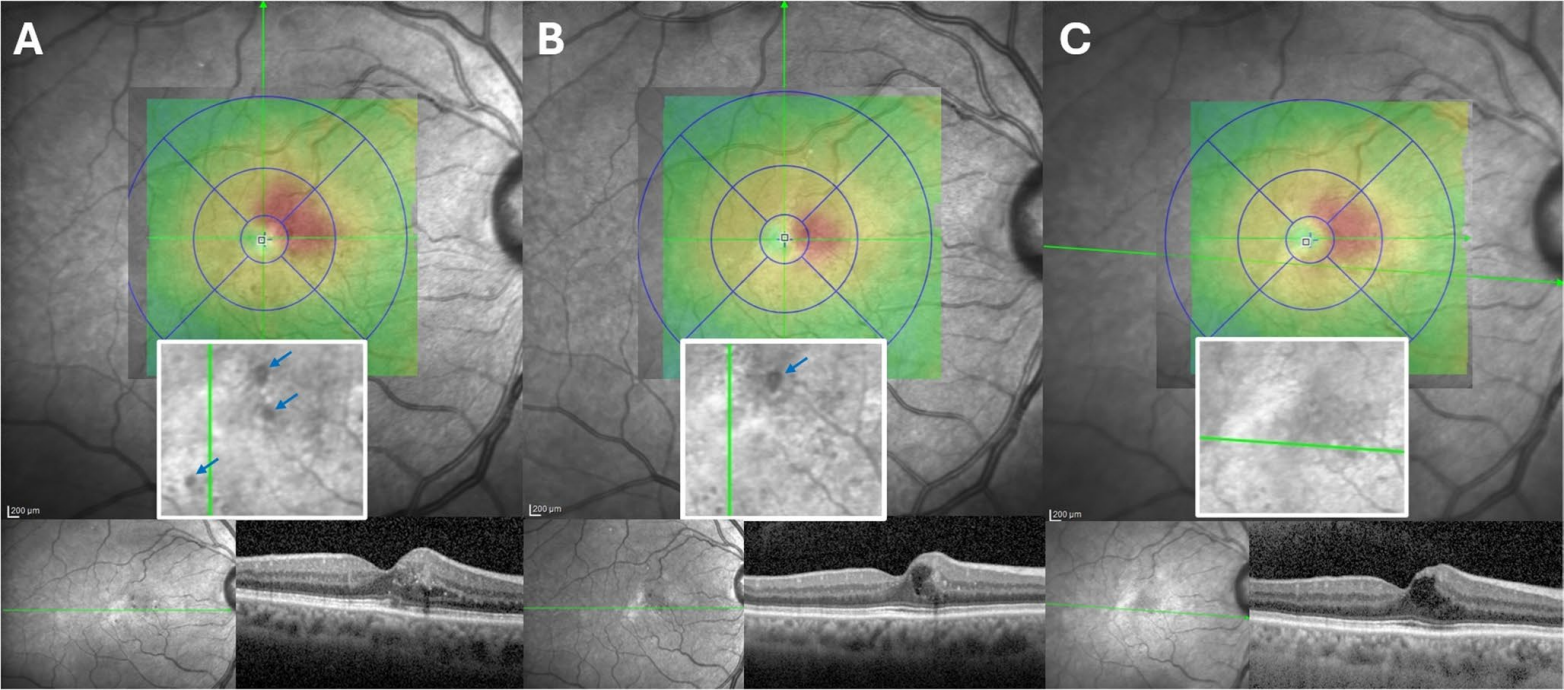
Demonstrative case of telangiectatic capillaries (TelCaps) remodeling over time. At presentation (**A**), three TelCaps were noted on infrared reflectance (IR), two superonasal to the fovea (126 and 114 μm), and one infero-temporal (104 μm) to it (blue arrows). On corresponding B-scans, there was center-involving diabetic macular edema (central subfield thickness [CST] = 321 μm). After 12 months of follow-up and three intravitreal injections of aflibercept (**B**), the last being 2 months prior, two TelCaps disappeared, and one remained with stable diameter on IR (121 μm). On B-scans, intraretinal fluid was now arranged in a chronic cystic appearance (CST: 338 μm). At the final follow-up of 21 months and after 2 more aflibercept injections (**C**), the last being 4 months prior, no TelCaps were detected on IR, and B-scans showed breakdown of the hyperreflective TelCap wall, with enlargement of the chronic cystic space (CST = 328 μm)

The average number of TelCaps per eye decreased to 1.12 ± 0.88 (*p* = 0.829) at 1-year and to 0.76 ± 0.83 (*p* = 0.04) at the last follow-up (Table 2). The TelCaps disappeared in 11 eyes (44%) on the final visit (Fig. 2), and new TelCaps were noted in 3 eyes (12%). The mean size of the TelCaps was significantly reduced at 1-year and the final follow-up compared to baseline on both IR (173.6 ± 74.1 μm [baseline], 112.3 ± 89.1 μm [1 year], 88.9 ± 90.9 μm [last follow up]), *p* < 0.001) and OCT B-scans (92.6 ± 42.5 μm [baseline], 71.6 ± 61 μm [1 year, *p* = 0.045], 60 ± 67.1 μm [final follow-up, *p* = 0.01]). The mean baseline size of the TelCaps that disappeared was significantly lower in IR images compared to those that persisted (149.8 ± 45.7 μm vs. 198.9 ± 90.3 μm, *p* = 0.049).

Compared to eyes with improved CST, eyes with worse CST on final follow-up had greater mean number of TelCaps on presentation (1.1 vs. 1.5, respectively), similar mean size of TelCaps on IR images (175 μm vs. 173 μm), received a lower mean number injections (5.6 vs. 4.3), and had more disappearance of TelCaps on the final visit (33% of eyes vs. 50% of eyes).

## Discussion

In this long-term follow-up of TelCaps in eyes with DME, we observed remodeling of the TelCaps with reduction in number and size over time, disappearance of the smaller lesions in almost half of the eyes by the final follow-up, and new lesions developing in a small subset of the eyes (12%). All findings were better detected and more accurately measurable on IR imaging compared to structural B-scans. On final follow-up, three-quarters of the eyes had unchanged or worse VA while two-thirds had increased CST. Accordingly, targeted laser therapy may possibly be a better strategy for treating those dynamic structures [21] and hence the need for accurate upright visualization.

In the original description of TelCaps, the authors utilized indocyanine green angiography (ICGA) to detect the characteristic late staining of the lesions [10]. They utilized a size cut-off of 150 μm to differentiate between microaneurysms and TelCaps but admitted that such a choice was arbitrary and that both lesions likely represent a continuum. ICGA is not routinely utilized for DME patients, is an invasive procedure that carries risks, and is not available in all settings, reflecting the need for a more accessible imaging tool to detect TelCaps. A recent Delphi study by the International Retinal Imaging Society proposed the use of the term “large retinal capillary aneurysms” to refer to any vascular anomalous lesion with a hyperreflective wall and a hyporeflective lumen and a diameter of at least 100 μm, suggesting that 100 μm may be a more appropriate cut-off [19].

Roh et al. successfully utilized IR images with corresponding OCT B-scans to detect TelCaps and monitor their response to laser therapy [16]. In a contemporary study on TelCaps of at least 100 μm in diameter in 101 eyes with DME or retinal vein occlusion, the authors compared the diagnostic sensitivity of different imaging modalities in reference to ICGA for the detection of TelCaps [11]. The highest sensitivity was seen with OCT B-scans (94%), followed by moderate sensitivity for En Face OCT (65%) and OCT-angiography (55%), and the lowest sensitivity was noted for colored fundus photography (39%). The sensitivity of IR images was, however, not studied. This is in line with the original report on TelCaps that showed poor utility of OCT-angiography in detecting TelCaps [10], most likely due to very slow or absent flow within the lumen of the lesions, confirmed on histopathological analysis [22]. We demonstrate the utility of standard OCT B-scans for lesion identification albeit with significant underestimation of lesion size in comparison to IR images (Table 2). We attribute that to the spacing between the OCT raster or radial scans that may miss the widest part of the lesion and, thus, not fully capture its true size. Accordingly, we recommend a high-density OCT scanning protocol as an adjunct to IR images for the detection of TelCaps in the absence of ICGA, but measurements are better reserved for IR images.

Our long term follow-up also provides novel insight into the dynamic nature of TelCaps. We observed remodeling of the TelCaps with gradual reduction in their count and size over time, and disappearance of the lesions in nearly half of the eyes after a mean follow-up of 4 years. Paradoxically, the CST showed an increase on the final follow-up. This discrepancy could be attributed to a more intensive treatment early on; indeed, within our cohort, most of the injections (75% of the mean injections) were undertaken in the first year, with later reduction in treatment frequency. This may imply that early and intensive injection therapy can result in regression of the TelCaps lesions, albeit not reflecting on the final CST and VA. An alternative explanation is that the reduction in TelCaps size and number is part of their natural history. A similar phenomenon is noted with the smaller microaneurysm lesions where new ones form and old ones disappear, and the rate by which such turnover occurs has been linked to the development and progression of DME [23]. This is supported by our observation that the mean size of the TelCaps that disappeared was significantly lower compared to those that persisted and that eyes with worse final CST had more TelCaps on presentation but more disappearance of TelCaps with follow-up.

The limited data on the features of DME associated with TelCaps seem to suggest an association with chronic persistent edema and a poor response to anti-VEGF therapy [10, 13, 15, 16, 24]. Conversely, a recent study from Japan analyzed the response of 12 eyes with DME associated with TelCaps to three loading doses of anti-VEGF injections [14]. The authors showed significant reduction in the number and size of TelCaps and in the CST, but with persistent edema surrounding the TelCaps. Indeed, it has been suggested that focal leakage in DME - unlike diffuse leakage - may not be VEGF-dependent [25]. Our study had a longer duration of follow-up, and the results agree with the remodeling seen in the TelCaps with injection therapy, although an association with poor therapeutic response cannot be concluded with the lack of a control group without TelCaps. One case report [24] and a case series of 3 patients [15] with persistent DME and TelCaps suggested a favorable response to intravitreal steroid therapy. In our subgroup analysis of eyes receiving intravitreal steroid implants, no significant difference was noted for VA nor CST on the final follow-up. Targeted laser photocoagulation has been shown to reduce the size of TelCaps and improve the DME [16, 26]. The TalaDME is an ongoing, multicentric, observer-masked randomized clinical trial comparing targeted laser photocoagulation combined with anti-VEGF therapy to anti-VEGF therapy alone in eyes with DME and TelCaps [12].

Limitations to the current study include the small sample size and the absence of a control arm to compare treatment response. The possibility of insufficient injection therapy may introduce bias in response assessment; a PRN approach is followed in our institution and the mean number of injections in our patients was about 5 injections. In that regard, the study also highlights the preferred treatment protocols and the real-world compliance issues with injection therapy for DME.

In conclusion, TelCaps are dynamic lesions that seem to remodel with long-term follow-up as captured on IR imaging and OCT B-scans. In this sample of eyes with DME associated with TelCaps treated with intravitreal injections, most cases still exhibited persistent DME after at least one year, suggesting that TelCaps may serve as a potential biomarker for refractory DME.

## Abbreviations/Acronyms

CST: Central subfield thickness
DM: Diabetes mellitus
DME: Diabetic macular edema
DRIL: Disorganization of the retinal inner layers
ETDRS: Early treatment of diabetic retinopathy study
EZ: Ellipsoid zone
IR: Infrared reflectance
LogMAR: Logarithm of the minimal angle of resolution
OCT: Optical coherence tomography
PRN: Pro re nata
SD: Standard deviation
TelCaps: Telangiectatic capillaries
VA: Visual acuity
VEGF: Vascular endothelial growth factor
